# A short- and long-term follow-up study of intersphincteric NASHA Dx implants for fecal incontinence

**DOI:** 10.1007/s10151-022-02645-6

**Published:** 2022-06-26

**Authors:** E. Ezra, J. M. Danielsson, W. Graf

**Affiliations:** 1 Department of Surgical Sciences, Uppsala University Hospital, 751 85 Uppsala, Sweden; 2 Department of Women’s and Children’s Health, Uppsala University Hospital, 751 85, Uppsala, Sweden

**Keywords:** Fecal incontinence, Bulking agents, Injection therapy, Pelvic floor

## Abstract

**Background:**

The bulking agent NASHA Dx injected into the submucosal layer is effective in the treatment of fecal incontinence (FI) at short-and medium-term follow-up but efficacy after injection in the intersphincteric location is unknown. The aim of this study was to determine the short- and long-term efficacy and safety of NASHA Dx injected into the intersphincteric location for FI.

**Methods:**

Patients were recruited from referrals to our Department for treatment of FI in November 2008–January 2010. Eligible patients were injected with 8 ml of NASHA Dx. Patients with a subtotal treatment effect were retreated after 2–4 weeks. The change in number of fecal incontinence episodes, the proportion of responders defined as at least 50% decrease in number of FI episodes and side effects were the main outcome measures.

**Results:**

Sixteen patients, 15 women and 1 man with a median age of 68, 5 (range 44–80) years and a median CCFIS of 15 (range 10–19) were included in the study. The median number of incontinence episodes decreased from 21.5 (range 8–61) at baseline to 10 (range 0–30) at 6 months (*p* = 0.003) and 6 (range 0–44) at 12 months (*p* = 0.05). The median number of incontinence episodes in the 11 patients completing the 10-year follow-up was 26.5 (range 0–68). The percentage of responders at 12 months and 10 years were 56% and 27%, respectively. Mild to moderate pain at the injection site was described by 69%. There was one case of mild infection, successfully treated with antibiotics and one implant had to be removed due to dislocation.

**Conclusions:**

NASHA Dx as an intersphincteric implant improves incontinence symptoms in the short term with moderate side effects and can be used alone or as an adjunct to other treatment modalities. Long-term efficacy was observed in 27%.

## Introduction

The prevalence of fecal incontinence (FI) is 1.4–19.5% in adults depending on the population investigated, data collection methods and definitions [1, 2]. Several authors agree on an overall community prevalence between 8 and 10% but since there is a paucity of data from certain age groups, minorities and low- and middle-income countries, the true prevalence is unknown [3]. Embarrassment leads many patients to avoid telling their caregiver about their FI, adding to the uncertainty about the true prevalence [4].

FI is a symptom with multiple etiologies and a majority of FI patients have more than one underlying abnormality [2, 5–8]. Moreover, FI can have a devastating impact on quality of life, affecting the patient physically, socially, emotionally and financially [2, 9]. A majority of seriously ill hospitalized patients deemed incontinence as a state equal to or worse than death, illustrating the considerable negative effect on autonomy [10].

The treatment of FI remains a challenge. Simillis et al. identified 37 different treatments for FI and long-term efficacy data are lacking [11, 12]. Injectable bulking agents have been used since 1993 with the aim of expanding and strengthening the upper part of the anal canal [13]. Injection of dextranomer in stabilized hyaluronic acid (NASHA Dx, Q-Med AB, Uppsala, Sweden), into the submucosal layer has been shown to be superior to sham treatment and associated with a decreased number of incontinence episodes [14]. Injection into the submucosal layer has advantages in terms of simplicity and immediate visual feedback. Injection into the intersphincteric layer allows a larger volume and, theoretically, creates a smoother and more symmetric constriction [15, 16]. To date, no study has evaluated NASHA Dx in this location and the aim of this study was to analyze the short- and long-term efficacy and safety of NASHA Dx injected into the intersphincteric location.

## Materials and methods

### Study design

This was a prospective, single center study with a short- (12 months) and a long term (10-year) follow- up.

The inclusion criteria were: FI with at least 2 episodes/week; symptom duration of at least 1 year, and failure of conservative therapy including at least two of the following therapies: dietary measures, fiber supplements, Loperamide medication, rectal enemas, pelvic floor muscle training or biofeedback. Further inclusion criteria were: age 18–80, written informed consent, availability for follow-up and expectance of full compliance with the protocol. Exclusion criteria were: active inflammatory bowel disease, total external sphincter defect at ultrasound and clinical examination, bleeding diathesis or anticoagulant therapy, rectal prolapse or intussusception, active anal sepsis, anorectal implants, recent anorectal surgery (within 1 year), rectal anastomosis, pregnancy, postpartum (1 year or less), breast feeding, mental disorder, immunodeficiency, pelvic irradiation, chronic pelvic pain, chronic anal fissure and symptoms consistent with outlet obstruction.

We excluded patients with prior implants to obtain a homogenous sample of implant treatment naive patients.

Patients were recruited from referrals to the Department of Surgery, Coloproctology Section, for treatment of FI in November 2008–January 2010. Eligibility was determined at the initial visit when written informed consent was obtained. Thereafter the following forms were completed by the patient: a 3-week diary (leakage of liquid and solid stool), Cleveland Clinic fecal incontinence score (CCFIS), Short Form Health Survey (SF-36) and a validated bowel function questionnaire [17]. Also, a case report form (CRF) including a self-assessment item was filled out at inclusion and during the primary trial visits i.e., up to 12-months. The patient returned after a screening period of 4 weeks and, if eligibility could be verified, treatment was administered (see below). Before treatment, all participants underwent a clinical examination, anal manometry and endoanal ultrasound as previously described [18]. Significant intussusception was excluded by palpation and rectoscopy [19].

The study, including the 12-month follow-up, was registered at Clinicaltrials.gov (NCT00971269).

The 10-year follow-up was initiated after a protocol amendment in which patients were contacted by mail and, if necessary, by phone. If they accepted participation written consent was obtained and the same questionnaires and 3-week diary as in the primary trial were sent out and completed.

The primary trial and the 10-year follow-up were approved by the institutional ethical review board (2008/066, 2020–01318).

### Treatment

According to the hospital routine prophylaxis for colorectal surgery, the patients ingested Metronidazole 2 g and Trimetoprim/Sulphametoxazol 160 mg/800 mg orally 4–8 h prior to treatment. The rectum was cleaned by means of rectal wash-out enema. The treatment was given in the left lateral position. Analgesia was achieved through a perineal block [20]. A 60 mm long injection needle was then inserted through the perianal skin and advanced upwards in the intersphincteric layer guided by an endosonography probe placed in the anal canal. When the tip of the needle was positioned at the lower level of the puborectalis muscle, the agent was injected over approximately 20 s at the 3, 6, 9 and 12 o’clock positions. Two milliliters were injected at each site for a total dose of 8.0 ml. The agent, NASHA Dx is a gel consisting of dextranomer microspheres in a stabilized hyaluronic acid-based gel of nonanimal origin (NASHA TM gel). It has been Conformitè Europëenne [European Conformity; (CE)] approved since 2006 and United States Food and Drug Administration (FDA) approved since 2010 under the trademark Solesta® for the treatment of FI. The dose at initial treatment was 8.0 ml in all patients and all patients were treated by the senior author (WG). After treatment, the patients could ambulate but remained at the clinic for 1 h in case of any adverse reactions. All patients were advised to avoid strenuous activities and pressure on the perineum for 4 weeks. After 2–4 weeks the patients were interviewed over the phone about remaining FI symptoms, any improvement and possible side effects. Patients were offered retreatment in case of a subtotal effect, defined as a decrease but not cessation in numbers of FI episodes, if there had been no side effects and if they were motivated to undergo a second procedure. Ten out of the 16 patients were retreated shortly after the telephone follow-up. Retreatment was identical to the first procedure except that the injection sites were in between the initial ones.

### Trial objectives and follow-up

The primary objective was the change in number of FI episodes at the 6-month, 12-month and 10-year follow-up versus baseline and the proportion of responders as markers of efficacy. Success i.e., clinically significant response was defined as a decrease of 50% or more in the number of FI episodes. Secondary objectives were: change in CCFIS, manometrical changes at 12 months, changes in deferring time for loose and solid stool and change in quality of life derived from SF-36 and the validated bowel function questionnaire and side effects.

The patients were followed up at 3, 6, and 12 months with an interview, clinical examination, rectoscopy, 3-week diary, CCFIS, SF-36, and the bowel function questionnaire. Treatment efficacy variables were only evaluated at 6 and 12 months and 10 years. In addition, at the 12-month follow-up, an anal manometry was performed. At the 10-year follow-up, the patients completed the 3-week diary, CCFIS, SF-36, and the bowel function questionnaire with the addition of the following questions: Since the 12-month follow-up, have you received any additional treatment for your fecal incontinence? If so, what kind of treatment and when? No clinical examination took place at the 10-year follow up.

### Statistical analysis

Values are presented as proportions or medians and ranges. The Wilcoxon matched pair test was used to assess paired differences for numeric variables and McNemar’s test for paired differences in proportions. Statistica software, version 13 (TIBCO, Tulsa, OK, USA) was used for statistical analyses. A *p*-value below 0.05 was interpreted as statistically significant.

## Results

### Patients

Sixteen patients with a median CCFIS of 15 (range 10–19) were included, 15 women and 1 man with a median age of 68, 5 (range 44–80) years (Table 1). At the 10-year follow-up, 2 patients had died; 2 could not be followed up because of morbidity (dementia and general weakness) and 1 patient declined to participate for unknown reasons. This left 11 patients to be evaluated in the long-term follow-up.

**Table 1:** Baseline demographic data

Patient	Age, years	Sex	Duration of FI years	Type FI	FI episodes/w	CCFIS	Previous anorectal surgery	Etiology	10-year follow- up
1	58	F	1–5	Combined	5	16	Rectopexy	Neurogenic	N
2	67	F	> 5	Passive	6	16		Neurogenic	N
3	69	F	> 5	Passive	10	13		Idiopathic	Y
4	45	F	> 5	Passive	13	15		Idiopathic	Y
5	74	F	> 5	Passive	20	17		Neurogenic	Y
6	51	F	> 5	Urgency	3	18	Hemorrhoidectomy	Neurogenic	N
7	80	F	> 5	Combined	3	15		Neurogenic	N
8	71	F	> 5	Combined	6	15		Neurogenic	Y
9	68	F	> 5	Combined	4	17		Neurogenic	Y
10	70	F	1–5	Passive	2	15		Neurogenic	Y
11	44	M	1–5	Passive	6	13		Idiopathic	Y
12	74	F	> 5	Passive	15	19		Neurogenic	Y
13	66	F	> 5	Urgency	8	10		Idiopathic	Y
14	69	F	> 5	Passive	31	18	Sphincteroplasty	Neurogenic	Y
15	70	F	1–5	Passive	8	16	Hemorrhoidectomy	Neurogenic	N
16	56	F	> 5	Passive	8	19	Sphincteroplasty	Obstetric	Y

*FI *fecal incontinence, *CCFIS* Cleveland Clinic Fecal Incontinence Score

### Results of all 16 patients completing 6- and 12-month follow-up

**Fig. 1:**
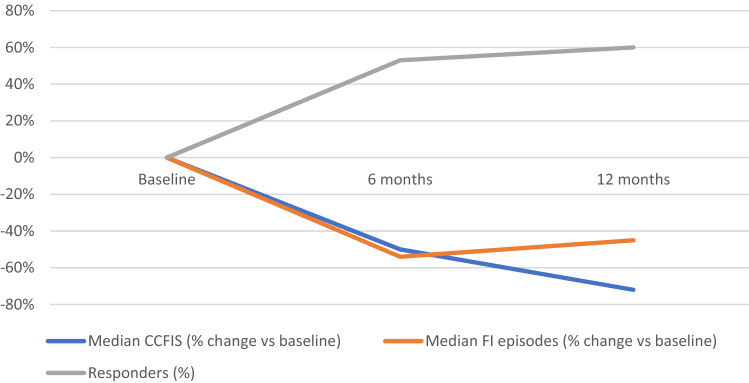
Changes in CCFIS and FI episodes as well as number of responders at 6 and 12 months. *CCFIS* Cleveland Clinic Fecal Incontinence Score, *FI* fecal incontinence

**Fig. 2:**
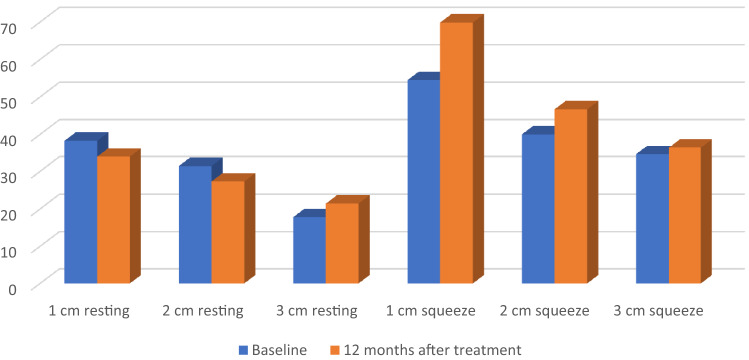
Manometry data at baseline and 12 months (mm Hg and centimeters from anal verge)

The median number of incontinence episodes for loose or solid stool decreased from 21.5 (range 8–61) at baseline to 10 (range 0–30) at 6 months (*p* = 0.003) and 6 (range 0–44) at 12 months (*p* = 0.05). Three patients were completely continent at 12 months, i.e., had no FI episodes and 2 patients had < 1 episode/week in the 3-week diary. A total of 9/16 (56%) were responders at 12 months. The median CCFIS decreased from 16 (range 10–19) at baseline to 8 (range 1–14) at 6 months (*p* = 0.0006) and increased slightly to 7.5 (range 3–16) at 12 months (*p* = 0.0007, Fig. 1). When compared with baseline no significant changes were seen for deferring time for loose stool at 6 months, median 2.5 min (range 0–20 min) (*p* = 0.06) or at 12 months, median 2 min (range 0–5 min) (*p* = 0.11). However, deferring time for solid stool was increased at 6 months, median 8 min (range 2–20) (*p* = 0.046) and 12-months, median 5 min (range 2–20 min) (*p* = 0.046). (Table 2). When comparing baseline and 12-month anorectal manometry data there were no significant differences in resting pressure at 1 cm (*p* = 0.20), 2 cm (*p* = 0.21) and 3 cm (*p* = 0.65) measured from the anal verge. There was a significant increase in squeeze pressure at 1 cm (*p* = 0.04) and 2 cm (*p* = 0.03) but not at 3 cm (*p* = 0.74, Fig. 2). There were no statistically significant changes in any SF-36 domain at 6 or 12 months when compared to baseline. When self assessing at 12 months all but one patient felt improved compared to baseline. Seven patients judged the result as excellent, 5 as good, 3 as fair and 1 as poor.

**Table 2:** Deferring time for loose and solid stool at 6 and 12 months for all 16 patients

	Median	Range	*P*-value
*Deferring time loose stool (minutes)*			
Baseline	0	0–5	N/A
6 months	2.5	0–20	0.06
12 months	2	0–5	0.11
*Deferring time solid stool (minutes)*			
Baseline	3	0–20	N/A
6 months	8	2–20	0.046
12 months	5	2–20	0.046

### Results of the 11 patients completing the 6-month, 12-month and 10-year follow-up

Two patients had received invasive treatment after the 12-month follow up (sacral neuromodulation in one and submucosal bulk therapy in one). None of the remaining 9 patients had received any invasive or surgical treatment for FI since the 12-month follow-up but all 11 patients had received different conservative treatments as listed in the inclusion criteria.

**Fig. 3:**
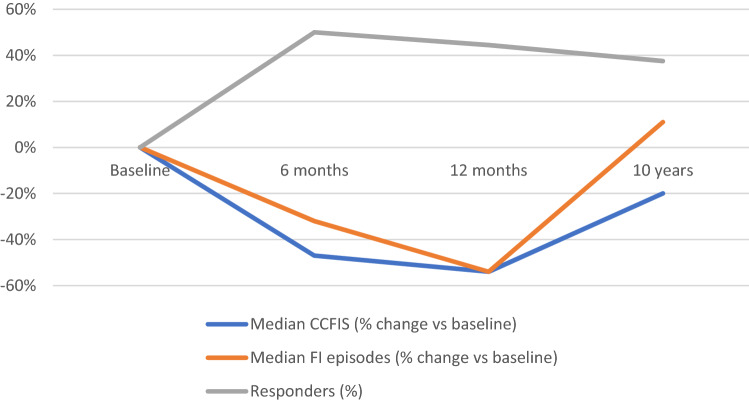
Changes in CCFIS and FI episodes as well as number of responders at 6 months, 12 months and 10 years. *CCFIS* Cleveland Clinic Fecal Incontinence Score; *FI *fecal incontinence

After 10 years, the median number of incontinence episodes was 26.5 (range 0–68) which, in this subgroup, was similar to baseline (25, range 6–92, *p* = 0.55). Two patients reported no incontinence episodes during the 3-week registration period. The proportion of responders had dropped to 27% compared to 54% at 12 months. The median CFIS decreased from 15 (range 10–19) at baseline to 8 (range 3–14) at 6 months (*p* = 0.005), to 7 (range 416) at 12 months (*p* = 0.005) and 12 (range 2–16) at 10 years (*p* = 0.02, Fig. 3). There were no significant changes in deferring time for loose or solid stool at the 10-year follow-up compared with baseline (Table 3). In this subset of patients i.e., the 11 patients that were followed up for 10 years quality of life showed a significant improvement in the SF-36 domain of social functioning at 12 months compared to baseline (*p* = 0.04). No significant changes were seen in this SF-36 domain at the 6 months (*p* = 0.25) or 10-year follow-up (*p* = 0.40). There were no significant changes in any other SF-36 domain.

**Table 3:** Deferring time for loose and solid stool at 6 months, 12 months and 10 years for the 11 patients included in the 10-year follow-up

	Median	Range	*P*-value
*Deferring time loose stool (minutes)*			
Baseline	0	0–5	N/A
6 months	2	0–20	0.23
12 months	3	0–5	0.14
10 years	0	0–20	0.50
*Deferring time solid stool (minutes)*			
Baseline	4	0–20	N/A
6 months	5	2–20	0.08
12 months	5	2–10	0.11
10 years	5	0–20	0.60

### Side effects

Pain during injection was described as mild in 8 cases, moderate in 3 and the remaining 5 felt no pain. A moderate anal ache lasting 2–4 weeks after injection was reported in 5 patients. In 1 patient, dislocation of an implant towards the perianal skin was observed, and this was treated with an incision and evacuation. In another patient a slight fever, a mild phlegmon and induration were treated successfully with oral antibiotics. One woman was diagnosed with a microscopic colitis after the 6-month follow-up. This event was not interpreted as related to treatment. In all cases the pain subsided and no patient experienced pain at 3 months or later. No side effects were reported at 12 months or 10 years.

## Discussion

To our knowledge, this is the first report of results after intersphincteric NASHA Dx injection and furthermore with a unique long- term follow up. Maeda et al. had a 5-year follow-up (6 patients) in 2007 and Guerra et al. presented a median follow-up of 7 years (19 patients) in 2015, but most long-term follow-up studies comprise 36 months or less [21–24].

The design of our study resembled previous studies implanting NASHA Dx in the submucosal layer, using a screening period that allowed the investigator to verify eligibility concerning severity of incontinence and comorbidity [14].

Intersphincteric implants create a different anatomical situation compared with submucosal deposits. The volume expansion is different when using intersphincteric injection, creating a smoother constriction and a need for a larger volume depending on an increased anatomical space. Submucosal Nasha Dx implants might slide in loose connective tissue planes [18].The risk for displacement might be different since the implants in our study are not in close proximity to the bowel content, but may still move in the connective tissue planes like infectious processes [25].

The injection procedure is more complicated, requiring infiltration analgesia, and is more difficult to perform compared to submucosal injections. All treatments were administered by the last author by ultrasound guidance confirming intersphincteric location at the anorectal junction. We recommend that clinicians performing this treatment are experienced in anal ultrasound. Therefore, it might be argued that intersphincteric NASHA Dx should be reserved for special circumstances i.e., when there is extensive scarring in the submucosa or when retreating patients who have already received a full dose in the submucosal plane. Patients should be counseled and informed about the risk of postimplant pain and complications.

In a randomized study using, PTP in the intersphincteric location, ultrasound guidance was associated with increased efficacy compared with clinically guided injections [26]. We believe ultrasound imaging is also of great value when injecting NASHA Dx intersphincterically: the needle can be seen when advanced and corrections can be made before injecting. The implant can also be visualized in real time during injection and, if the location is poor, the injection can be stopped and resumed in the correct position.

The proportion of responders at 12 months (56%) is consistent with other publications and there was a numerical but not statistically significant decrease in the number of incontinence episodes [14, 24].

Many authors will argue that bulking therapy is primarily indicated when treating internal anal sphincter deficiency, i.e., decreased resting pressure, but our material shows an increase only in the squeeze pressure [2, 27]. Bartlett et al. found an increase in both resting and squeeze pressure after intersphincteric bulking therapy [15]. Some authors have seen the same effect after submucosal injections while others have seen no effect on anal pressure after bulking therapy [28, 29]. These findings possibly indicate augmentation of both the internal and external sphincter. The intersphincteric location and the larger volume might cause the increase in squeeze pressure. We have no clear explanation why resting pressure was unaltered but a change might be seen in a larger patient series.

The increased deferring time for solid stool may also indicate an enhanced function of the external anal sphincter.

The reduction in CCFIS and positive self-reported outcomes are compatible with the findings of La Torre et al. [24].

A striking finding in our 10-year follow-up is the persistently decreased CCFIS, despite a reduction of the number of responders, no reduction in the number of incontinence episodes or increase in deferring time compared to baseline. In the long-term follow-up, the proportion of responders had dropped to 27%. In light of this drop it is surprising that only 2 out of the 11 patients had received additional invasive treatment.

The frequency and severity of the side effects are in line with published data describing the side effects as mild to moderate [14, 24, 30]. We consider the antibiotic prophylaxis and bowel cleaning as important measures to minimize infections.

There are several challenges in evaluating and comparing bulking treatment for FI. Firstly, there is no international consensus concerning the definition of FI in general, or concerning the definition of FI subtypes, including the impact on quality of life. Secondly, as previously stated, FI is a symptom with multiple etiologies indicating that most treated cohorts are heterogenous [2, 5–8]. Finally, there are at least 11 bulking agents on the market, and site, volume, route and method of injection differ from study to study [9, 31]. A 2013 Cochrane review concluded that the quality is poor in most trials, adding to the challenge [12].

The main limitations in our study are its small size and the heterogeneity of the patients including the different conservative treatment modalities received prior to inclusion and the absence of a clinical examination at the 10-year follow- up. However, many patients are referred to us from remote hospitals and considering our patients age and general condition, we did not deem it justifiable to bring them in for an examination at the long term follow up. Relying on a set of forms has its inherent weaknesses and does only provide a 3-week snapshot of the patient’s FI.

Our findings indicate that general coping strategies seem to play an important role in persistently lowering the CCFIS over time.

The ideal long-term follow-up and treatment of FI is individualized and based on regular clinical examinations and interviews [5]. The aim should be to characterize the FI according to future acknowledged international standards that include the impact on quality of life [2, 9]. Treatment should be tailored with multiple modalities that include empowering coping strategies [32].

Informing the patient that FI is a chronic condition with multiple causes and remedies over time might be a more realistic approach than promising cure. It might also lower the threshold for the patient to seek additional help in case of poor treatment outcome or recurrence [4].

## Conclusions

This study suggests that NASHA Dx as an intersphincteric implant results in a definite improvement of incontinence symptoms with moderate side effects in the short term. The long-term follow-up indicates a diminished efficacy.

We believe intersphincteric injection can be a part of a multimodal treatment approach, especially when submucosal injection is unsuitable. It can alleviate symptoms, augment coping mechanisms and bridge the gap between conservative treatment and surgery [27].
